# EHMTI-0296. Estimating prevalence and burden of major disorders of the brain in Nepal: methodology of a nationwide population-based study

**DOI:** 10.1186/1129-2377-15-S1-B21

**Published:** 2014-09-18

**Authors:** K Manandhar, A Risal, T Steiner, A Holen, R Koju, M Linde

**Affiliations:** 1 Department of NeuroscienceFaculty of Medicine, Norwegian University of Science and Technology Trondheim, Trodheim, Norway; 2 Department of Medicine, Dhulikhel Hospital Kathmandu University Hospital, Dhulikhel, Nepal

## Introduction

The major disorders of the brain are public health problems globally also prevalent in the low and middle income countries where they are poorly described.

## Aim

The aim this paper was to report how standard method developed by Lifting The Burden for population-based study was carried out in Nepal, a country with rather unusual physiographic challenges. The final goal of the research was to find the prevalence and burden of the major disorders of the brain – headache, anxiety, and depression in the adult population.

## Methods

Expert group collaboration was initially the method used to arrive at the instruments to be employed. Subsequently, pre-pilot and pilot studies were made to test and modify the final version. The study was an unannounced door-to-door survey to cover all parts of the country. Fifteen representative districts out of 75 in the country were investigated. Furthermore, households in the cluster were selected by using a “spin the bottle” method. Face-to-face interviews were carried out and involved one randomly selected adult family member. The structured questionnaire, originally developed by Lifting The Burden, with the addition of the Hospital Anxiety Depression Scale and the Eysenck’s neuroticism scale was used. These additional scales were translated into Nepali and psychometric validation was also done.

## Results

Among 2,210 contacted households, 2,109 were eligible for the study, and 2,100 (99.6%) adults from eligible households finally participated in the study.

## Conclusion

The reported method and the extended and translated HARDSHIP questionnaire were found adequate for the Nepali context.

No conflict of interest.

